# Evaluation of the exit screening policy among travelers arriving from Asian and pacific nations

**DOI:** 10.1186/s12879-024-09327-8

**Published:** 2024-05-02

**Authors:** Shiqi Liu, Asami Anzai, Hiroshi Nishiura

**Affiliations:** Kyoto University School of Public Health, Yoshidakonoe cho, Sakyo-ku, Kyoto City, 6068501 Japan

**Keywords:** Severe acute respiratory syndrome coronavirus 2, Border control, Quarantine, Travel, Statistical estimation, Effectiveness, Southeast Asia, Western Pacific

## Abstract

**Background:**

The Japanese government has instituted border control measures against COVID-19, including entry and exit screening of people arriving from overseas. We sought to evaluate the effectiveness of the exit screening policy in Japan in reducing the risk of importing COVID-19 cases among travelers from Asian and Pacific countries.

**Methods:**

The study period was stratified based on the timing of exit screening: (i) the control period (the pre-exit screening period from 25 October 2020 to 16 January 2021), (ii) the time period with the Alpha variant from 17 January to 10 April 2021, and (iii) the time period with the Delta variant from 2 May to 2 October 2021. Incidence data in the countries of origin were used to adjust for the risk of infection among travelers. The positivity rate of entry screening in Japan was compared among the three different study periods, adjusting for the risk of infection in the country of origin.

**Results:**

The adjusted relative risk of positivity was greatly reduced and substantially below the value of 1 during the Alpha variant period compared with the control period. Although the relative risks increased when comparing the Delta variant period against control, the estimate remained below 1, except for among travelers from India and Myanmar. The relative risk reduction was greatest in high-income countries, with estimates of 100% and 96% risk reduction during the Alpha and Delta variant periods, respectively, followed by upper-middle-income countries with estimates of 90% and 76%, respectively.

**Conclusions:**

Even in the presence of the Alpha and Delta variants, exit screening clearly reduced the risk of infection among travelers arriving from Asian and Pacific nations. As the testing relies on the country of origin, the effectiveness varied greatly by the socioeconomic income status and epidemiological situation of those countries. Test standardization and quality assurance may be required in low- and middle-income countries.

**Supplementary Information:**

The online version contains supplementary material available at 10.1186/s12879-024-09327-8.

## Background

The COVID-19 pandemic has led to a substantial decrease in the volume of travel, marked by a significant reduction in airline transportation resulting from the closure of multiple country borders and cancellation of international flights for safety reasons. Simultaneously, various countermeasures for border control have been introduced, aiming to ensure the absence of infection among arriving passengers (as well as departing passengers), while satisfying the need for international travel [1–4].

From March 2020 to December 2022, Japan adopted border control measures; of these, entry screening was carried out primarily at designated international airports—Tokyo-Narita, Tokyo-Haneda, and Kansai, which were gradually increased including Chubu (from June 2020), Fukuoka (from November 2020), Okinawa (from August 2022), and Hokkaido-Chitose (from December 2022). All incoming passengers, including Japanese citizens, were required to undergo testing upon arrival, via real-time reverse transcription-polymerase chain reaction (RT-PCR) testing, and from late July 2020 also by quantitative antigen testing of saliva specimens, as part of the entry screening process. Beginning in September 2020, Japan initiated a temporary removal of the prohibition on the re-entry of foreign nationals, and at the same time mandated exit screening, i.e., submission of a certificate of negative RT-PCR testing that had been conducted within 72 h of departure [5]. Except for tuberculosis for selected nations and for obtaining residence permit, COVID-19 was the first disease to which exit screening was legally imposed. On 20 December 2020, the detection of the Alpha variant (B.1.1.7) in a traveler arriving from the United Kingdom triggered the swift imposition of further measures, including from 27 December 2020 the requirement for Japanese nationals arriving from the United Kingdom (UK) being required to provide a negative pre-departure test certificate [6]. Given the further development of the epidemic, on 13 January 2021, all passengers, regardless of nationality and origin of departure, were mandated to provide a negative pre-departure test certificate when entering Japan [7]. Detailed time-dependent changes in entry and exit screening measures are described in Table 1.

**Table 1:** Description of airport screening policy and border control measures against COVID-19 from September 2020 to March 2022 in Japan

Calendar time (Date/Month/Year)	Event description
1/9/2020	Issued temporary lifting of foreign national re-entry prohibition, but imposing mandatory pre-departure negative RT-PCR testing submission (i.e. exit screening)
1/10/2020	Issued temporary permission for foreign national new entry with mandatory pre-departure negative RT-PCR testing submission (exit screening)
1/12/2020	Detection of the first case infected with Alpha variant from the UK at an airport
2/12/2020	Health follow-up via questionnaires mandated for individuals who have at least a 14-day stay history in Hubei Province or Zhejiang Province, China
24/12/2020	Issued prohibition on new entries (non-visa entry) among UK nationals
26/12/2020	Started mandatory quarantine at a facility nearby airport with post-hoc testing at Day 3
27/12/2020	Issued pre-departure negative RT-PCR testing certificate requirement (exit screening) for returning Japanese from the UK
28/12/2020	Suspension of new entries for foreign nationals from all nations
13/1/2021	Compulsory pre-departure negative RT-PCR testing certificate requirement for all travelers (including Japanese), regardless the country of origin
28/3/2021	Detection of the first case infected with Delta variant from India at an airport
14/5/2021	Issued prohibition on re-entry travelers from India, Nepal, and Bangladesh
4/6/2021	Started six-day quarantine at a facility nearby airport with post-hoc testing at Day 3 and Day 6
20/9/2021	Permission granted for re-entry travelers from India, Nepal, and Bangladesh
1/10/2021	Mandatory vaccination passport (submission certificate of vaccination) requirement for all travelers (otherwise, entry screening followed by quarantine)
8/11/2021	Resumption of new entries for foreign nationals from all nations
30/11/2021	Suspension of new entries for foreign nationals from all nations
30/11/2021	Issued cancellation of vaccination passport policy (submission certificate of vaccination)
1/3/2022	Resumption of new entries among foreign nationals from all nations
26/9/2022	Cancellation of all entry screening testing with regard to COVID-19
27/12/2022	Resumption of COVID-19 entry screening for travelers arriving from China (excluding Hong Kong and Macau) or having the history of stay during the past 7 days
9/1/2023	Requirement of a 72-hour pre-departure certificate and entry screening for passengers from Macau
28/4/2023	Cancellation of pre-departure certificate and vaccination certificate for all incoming passengers. Entry screening for symptomatic individuals from China and other countries was carried out.

While many countries implemented mandatory exit screening [8], little research has been done to quantify the effectiveness of such screening measures. Few studies have employed epidemiological or simulation-based methods to assess exit screening performance. According to one simulation study, anterior nasal PCR testing within 3 days of departure resulted in a 36% reduction in the total number of infectious days (95% confidence interval [CI]: 29–41%) [9]. Another study suggested that testing 3 days before travel led to a 10–29% decrease in the risk of transmission, while testing on the day of travel resulted in a more substantial reduction, by 44–72% [10]. Additionally, rapid antigen testing immediately before departure was associated with a decrease in post-arrival transmission by 37.4–46.7% [11]. From 20 March to 3 September 2022, pre-departure testing ≤ 1 day was linked to a 52% reduction in post-arrival diagnosis of severe acute respiratory syndrome coronavirus 2 (SARS-CoV-2) in the United States (US) [12]. A study in the UK indicated that pre-flight testing within 24 h reduced the entry of infectious travelers into the community, with a relative risk of 0.69 compared to scenarios with no testing before departure or after arrival [13]. However, few studies have drawn their conclusions from empirically observed exit screening data, and even with the same exit screening strategy (within 24 h), there are likely variations in the performance of exit screening. Also, many relevant studies have focused on domestic travelers or those within the European Union (EU) or from the US, indicating the need for a comprehensive analysis that could potentially resolve the shortage of evidence from other parts of the globe.

Japan is an East Asian country in the Western Pacific region with close connections and communications to neighboring nations in the Western Pacific and Southeast Asia. Using the entry screening dataset of passengers from Asia-Pacific countries and analyzing the local epidemiological dataset at the origin before and after the exit screening measure, we aimed to evaluate the effectiveness of the exit screening policy in Japan from October 2020 to October 2021.

## Methods

### Brief description of the border control policy in Japan

**Fig. 1:**
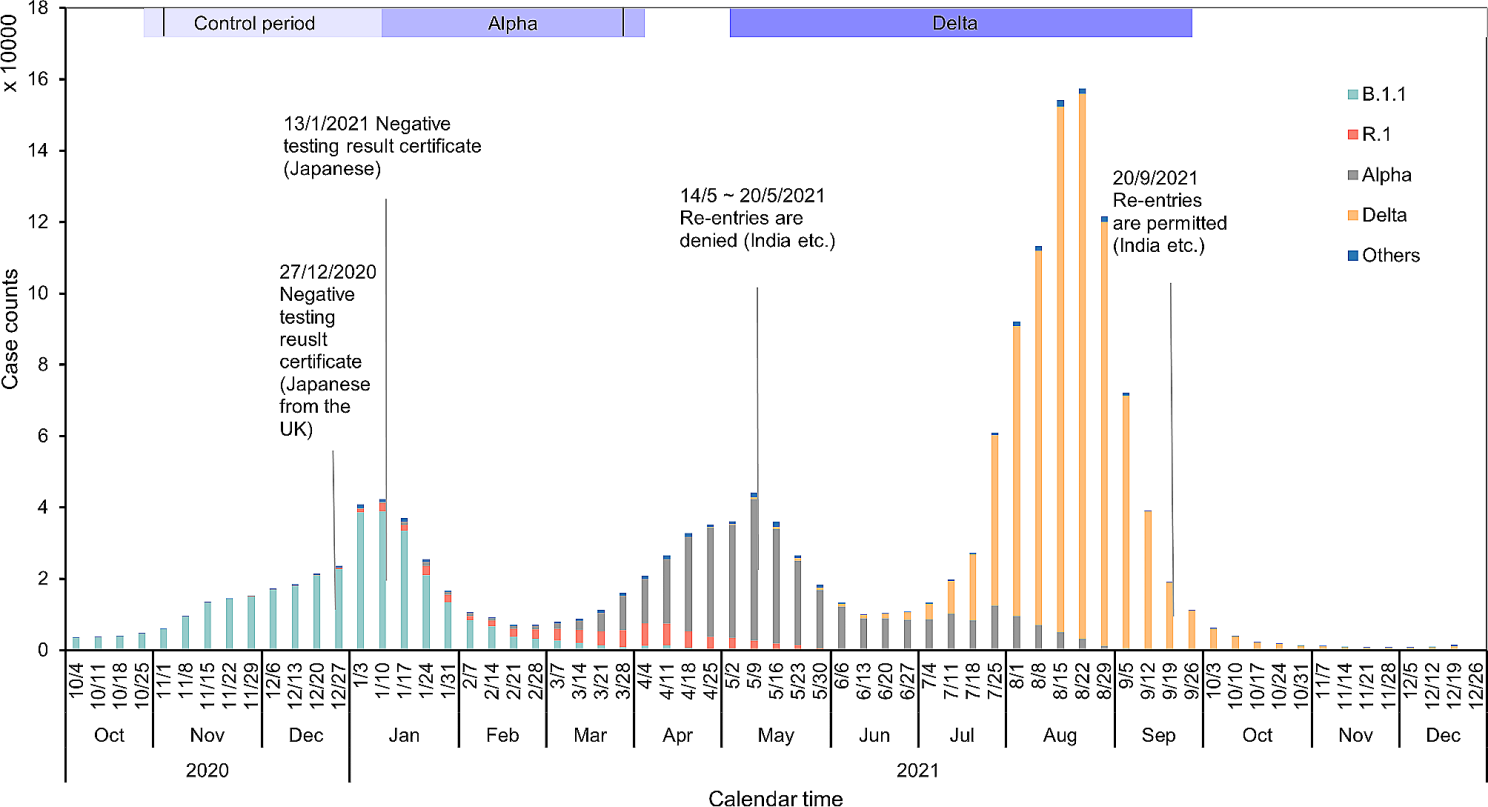
Epidemic dynamics of COVID-19 in Japan, 2020–21. Border control policy changes were overlaid with variant-proportionated domestic weekly cases in Japan from October 2020 to December 2021. The purple gradient bar at the top represents, from left to right, the control period, the Alpha variant period, and the Delta variant period. The areas between the vertical lines indicate the study periods among foreign travelers. The different colors on the bar represent the proportions of cases attributed to different variants of concern owing to local transmission in Japan

Initially, the exit screening policy was different between Japanese citizens and non-Japanese. Starting from 1 September 2020, foreign nationals entering Japan were required to undertake exit screening, presenting a certificate of negative RT-PCR or quantitative antigen testing result carried out within 72 h of departure. Japanese citizens were initially free from the exit screening requirement, but this same requirement later began to be enforced from 13 January 2021 (Fig. 1). In addition, genome sequencing of all positive samples was carried out. The first diagnosis of the Alpha variant, in a traveler from the UK, was made at the airport on 1 December 2020 [14], while the initial diagnosis of the Delta variant (B.1.617.2), in a traveler from India, took place on 28 March 2021 [15] (Table 1).

While the general exit screening policy timeline has been outlined above and in Table 1, there were minor variations among foreign travelers from Asian and Pacific countries. Given the limited risk of infection compared with other countries, non-Japanese from Australia, China, and South Korea, among others, etc. were exempted from presenting pre-departure test result certificates for an 11-week period from 1 November 2020 until 13 January 2021. Because of their very limited number, foreign travelers from Myanmar were required to undergo exit screening only beginning 1 November 2020. For similar reasons, non-Japanese citizens from Cambodia, Fiji, Laos, Mongolia, and Sri Lanka were only required to provide the certificates starting from 13 January 2021 (Supplementary Figure S1).

### Epidemiological dataset

All arriving passengers entering Japan, regardless of nationality and origin of departure, were required to undergo immediate RT-PCR or antigen testing upon arrival. The results of the entry screening tests conducted between 4 October 2020 and 23 April 2022 were publicly announced by the Ministry of Health, Labour, and Welfare. Because publicly available entry screening data starts from the time at which exit screening was already underway for non-Japanese travelers, we used entry screening results among Japanese nationals. This provided a control period with no exit screening requirement, i.e., between 4 October 2020 and 13 January 2021. The numbers of tests and positive test results were recorded weekly by nationality (i.e., for Japanese and other nationals) for air flights originating from a certain country [16].

In the present study, the selection of countries in the Western Pacific and Southeast Asian regions followed the World Health Organization classification [17], involving 11 countries in the Southeast Asia region and 37 countries and areas in the Western Pacific region. Of these, 19 countries with zero Japanese travelers and 20 countries with a limited number of travelers and without any positive cases at all were excluded from the analysis. In total, nine countries were subject for country-specific analysis, i.e., China, South Korea, Indonesia, Vietnam, Malaysia, the Philippines, Myanmar, India, and Bangladesh.

In addition to entry screening data, we examined the weekly incidence of COVID-19 in these nine countries, derived from national surveillance records. The daily number of confirmed cases were retrieved from the repository of the Center for Systems Science and Engineering at Johns Hopkins University [18]. The incidence level at the origin country was regarded as reflecting the prior probability of infection among travelers.

### Comparison across time periods

According to the timing of exit screening among Japanese travelers, we defined a control period spanning the 12 weeks from 25 October 2020 to 16 January 2021. The subsequent 12 weeks, from 17 January 2021 to 10 April 2021, were defined as the time period dominated by Alpha variant, coinciding with the enforcement of mandatory exit screening for all Japanese nationals from 13 January 2021 in response to the emergence in the UK [7]. Because exit screening became mandatory for Japanese from 13 January 2021, the week that contains 13–16 January might not strictly be regarded as control. Thus, we also examined the results using an alternative control period for 11 weeks, i.e., from 25 October 2020 to 9 January 2021. Except for India and Bangladesh, the time period dominated by the Delta variant was defined as lasting from 2 May to 2 October 2021 (Fig. 1). As the first Delta variant-infected traveler from India was diagnosed on 28 March 2021, the time period for India dominated by the Delta variant was assumed to have been already established earlier, and the predominant time period of Delta variant was defined as lasting from 11 April to 2 October 2021.

For this evaluation, we calculated unadjusted and adjusted prevalence ratio comparing positivity among travelers between two time periods, i.e., Alpha vs. control and Delta vs. control. As an adjustment factor, the prevalence at the origin country was calculated from the local incidence data obtained from the repository [18] for each period followed by its division by the population estimate in 2021. The prevalence was obtained as a convolution of the incidence and the survivorship function of virus positivity [19]. The relative risk of positive testing among those undertaking exit screening was calculated as the ratio of the adjusted positivity during the period dominated by the Alpha or Delta variant to the adjusted positivity during the control period. The adjusted positivity was defined as the unadjusted positivity (i.e., positivity from entry screening) divided by the cumulative incidence of the country of origin. The effectiveness of exit screening was derived from relative risk reduction, or the one minus adjusted prevalence ratio. The 95% CIs of positivity rates were determined using the Wilson score method. While depicting the relative risk and unadjusted prevalence ratio, their 95% CIs were calculated using the Wald confidence interval.

### Sensitivity analysis

In the baseline analysis, the length of the control period without exit screening was fixed as a 12-week long period. To address the uncertainty potentially associated with this length, we also computed the effectiveness of exit screening, varying its length from 9 to 15 weeks.

### Grouped analysis and analysis among foreign travelers

Among countries with non-zero data, we performed a grouped analysis by income level, i.e., the classification of subgroups following the World Bank country classification [20]. In the Southeast Asia and Western Pacific regions, most countries fall into the high-income, upper-middle-income, and lower-middle-income categories. Although there are 48 countries and areas in these regions, not every country was included in the analysis; countries with fewer than 10 weekly Japanese travelers or no Japanese travelers for consecutive weeks were excluded from the selection. Countries that were classified as high-income were Australia, South Korea, New Zealand, and Singapore. Similarly, upper-middle-income countries included China, Fiji, Indonesia, Malaysia, and Thailand. Lower-middle-income countries included Bangladesh, India, Cambodia, Lao PDR, Sri Lanka, Myanmar, Mongolia, Nepal, the Philippines, and Vietnam. Positivity and effectiveness of exit screening were computed for those three groups, using weighted positivity by travel volume.

We performed the same analysis among foreign travelers, focusing on countries that had no requirement for exit screening for non-Japanese citizens during the control period from 1 November 2020 to 13 January 2021. The time periods dominated by the Alpha and Delta variants are dealt with as the same as the analysis among Japanese travelers. This analysis was able to be conducted for China, South Korea and Sri Lanka.

## Results

**Fig. 2:**
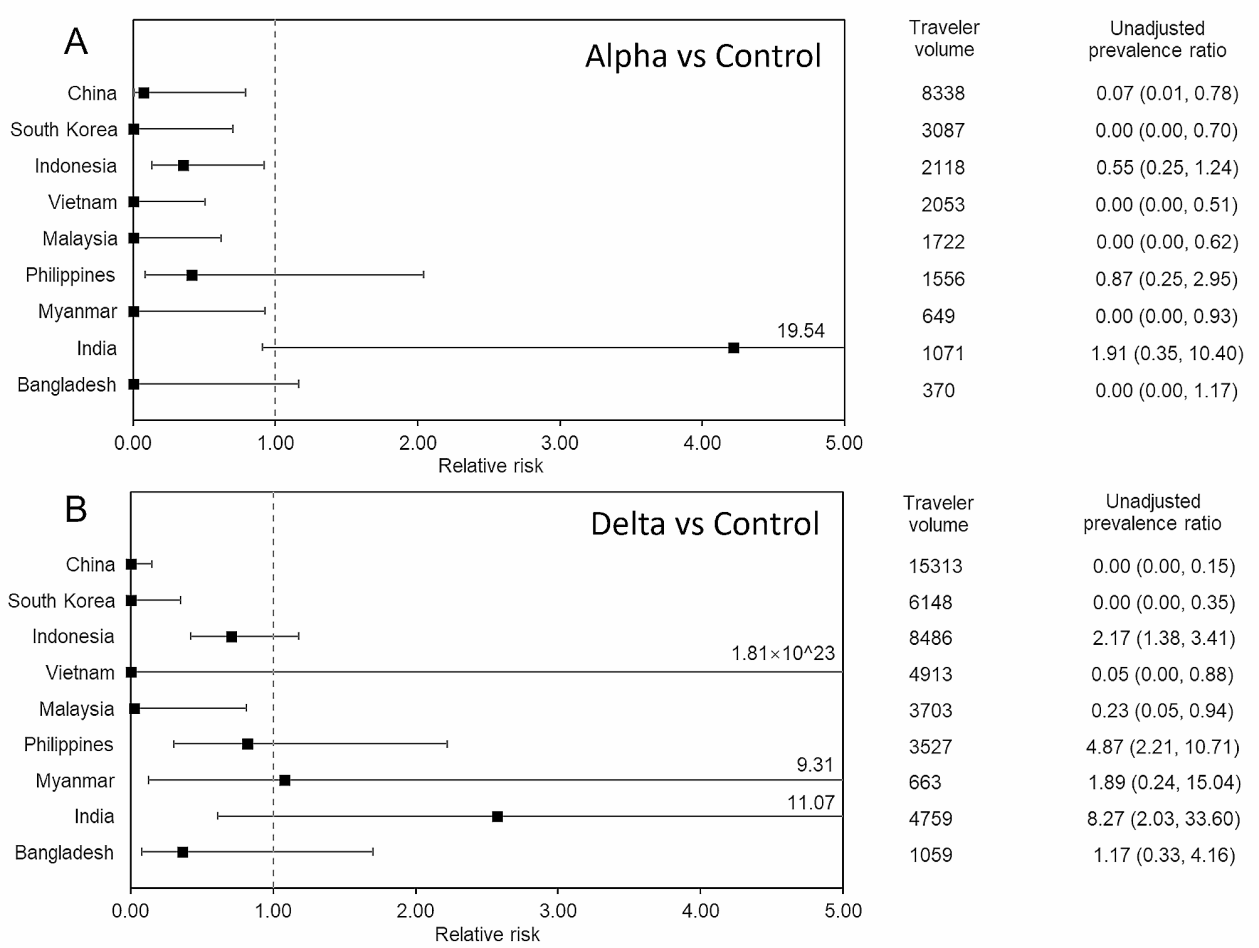
Relative risks of COVID-19 among Japanese travelers returning from nine countries during the Alpha and Delta periods compared with the control period, 2020–21. Relative risks of nine countries during (**A**) the Alpha variant period and (**B**) the Delta variant period. The risk in these periods with exit screening was compared against the risk during the control period without exit screening practice among Japanese nationals. For both periods, the dashed line signifies a relative risk value of 1 (i.e., no indication of the effectiveness of exit screening). Upper bound values among certain countries are shown as numerical numbers on the whisker. In the unadjusted prevalence ratio columns, values inside parentheses represent the corresponding 95% confidence intervals derived from binomial distribution

Figure 2 displays the relative risk of positivity during the Alpha and Delta variant dominated periods compared against the control period among the nine countries that were included. When the Alpha variant was dominant (Fig. 2A), the relative risk was generally below one except for India. Five countries, i.e., South Korea, Vietnam, Malaysia, Myanmar, and Bangladesh, showed the expected value of relative risks to be zero. When the Delta variant replaced others (Fig. 2B), the relative risk of infection among Japanese travelers from India and Myanmar exceeded one, although the 95% CI of Myanmar spans from 0.12 to 9.31, and thus, the statistical significance cannot be demonstrated. Conversely, the relative risks for South Korea and China were zero. Among the remaining countries, relative risks were elevated compared with the comparison between the Alpha variant and control period, and in fact, the unadjusted prevalence ratio when the Delta variant was dominant exceeded the value of one among travelers from Indonesia, Philippines, Myanmar, India and Bangladesh. Even when the length of control period was varied from 9 to 15 weeks, our sensitivity analysis showed that these results remained unchanged (Supplementary Figure S2). The qualitative patterns of orders for relative risk reduction were maintained from Fig. 2. During the period in which the Delta variant was dominant, results consistent with Fig. 2 were again observed, and India showed an absence of reduced risk during the period when exit screening was mandated (Supplementary Figure S3). Even when we removed the week that contained 13–16 January 2021 from control period, our qualitative findings were not altered (Supplementary Figure S4).

**Fig. 3:**
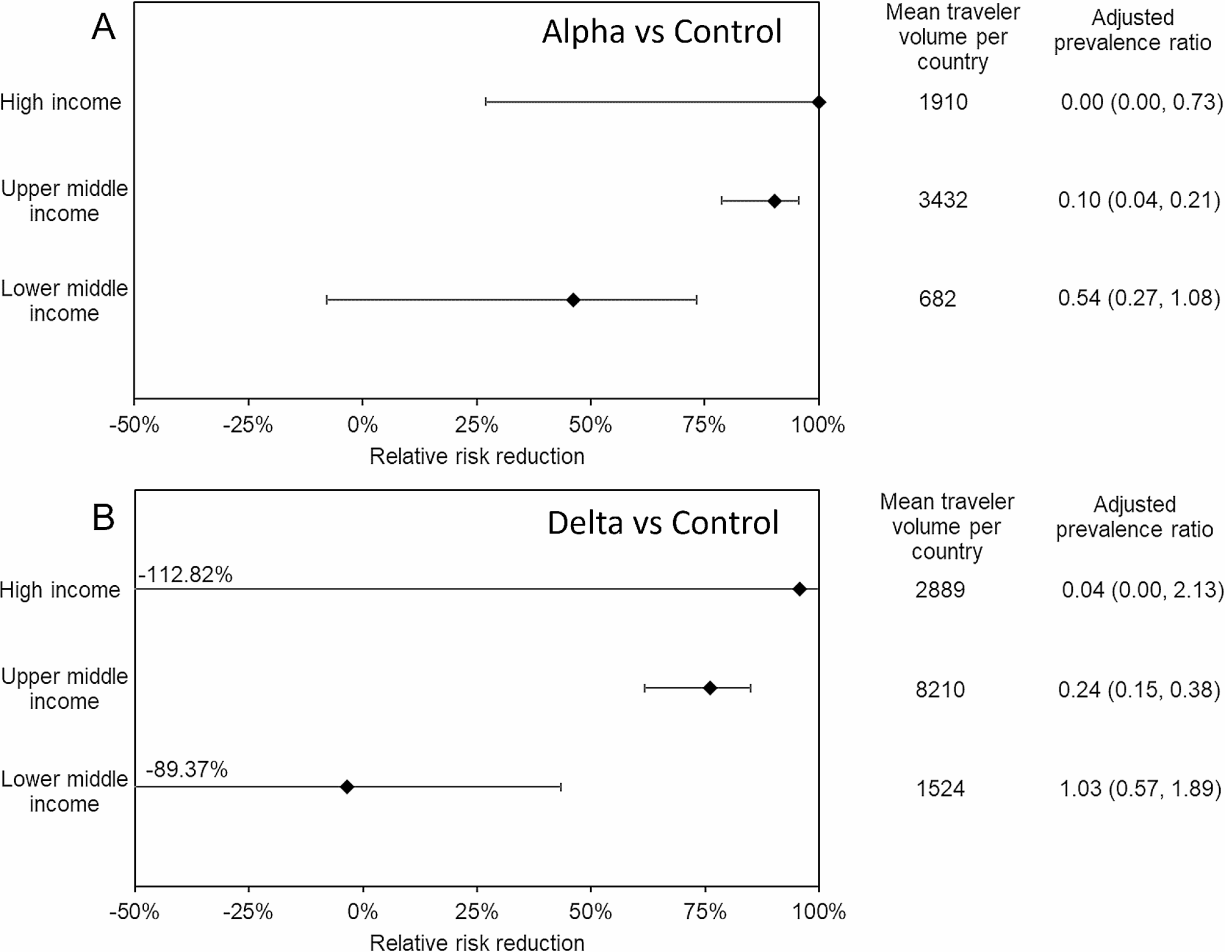
Relative risk reductions of COVID-19 among returning Japanese by country income level category. Relative risk reductions during (**A**) the Alpha variant period and (**B**) the Delta variant period among Japanese travelers returning from high-income, upper-middle-income, and lower-middle-income countries are shown. Extremely small lower bound values for certain countries are shown on the whisker. Within the adjusted prevalence ratio column, values in parentheses indicate the corresponding 95% confidence intervals derived from binomial distribution

Figure 3 illustrates the relative risk reduction via grouped analysis by income levels, comparing the Alpha and Delta variant periods against the control period. When the Alpha variant was dominant (Fig. 3A), the highest relative risk reduction was observed among high-income countries at 100% (95% CI 26.9–100), followed by upper-middle-income countries at 90.4% (95% CI 78.7– 95.7). When the Delta variant replaced others (Fig. 3B), the relative risk reduction decreased among all three groups. The high-income group experienced a slightly decreased relative risk reduction at 95.9% (95% CI -112.8–100), followed by upper-middle-income countries at 76.1% (95% CI 61.9– 85.0). Although the 95% CI of the high-income group crossed 0%, and thus, the statistical significance cannot be established, the expected values by income status were consistently ordered. The relative risk reduction of lower-middle-income countries was expected to be below 0%, implying that the risk of infection among travelers returning from those countries was not reduced by exit screening. For both periods, the volume of travelers was greatest among those returning from upper-middle-income countries, followed by those returning from high-income countries.

**Fig. 4:**
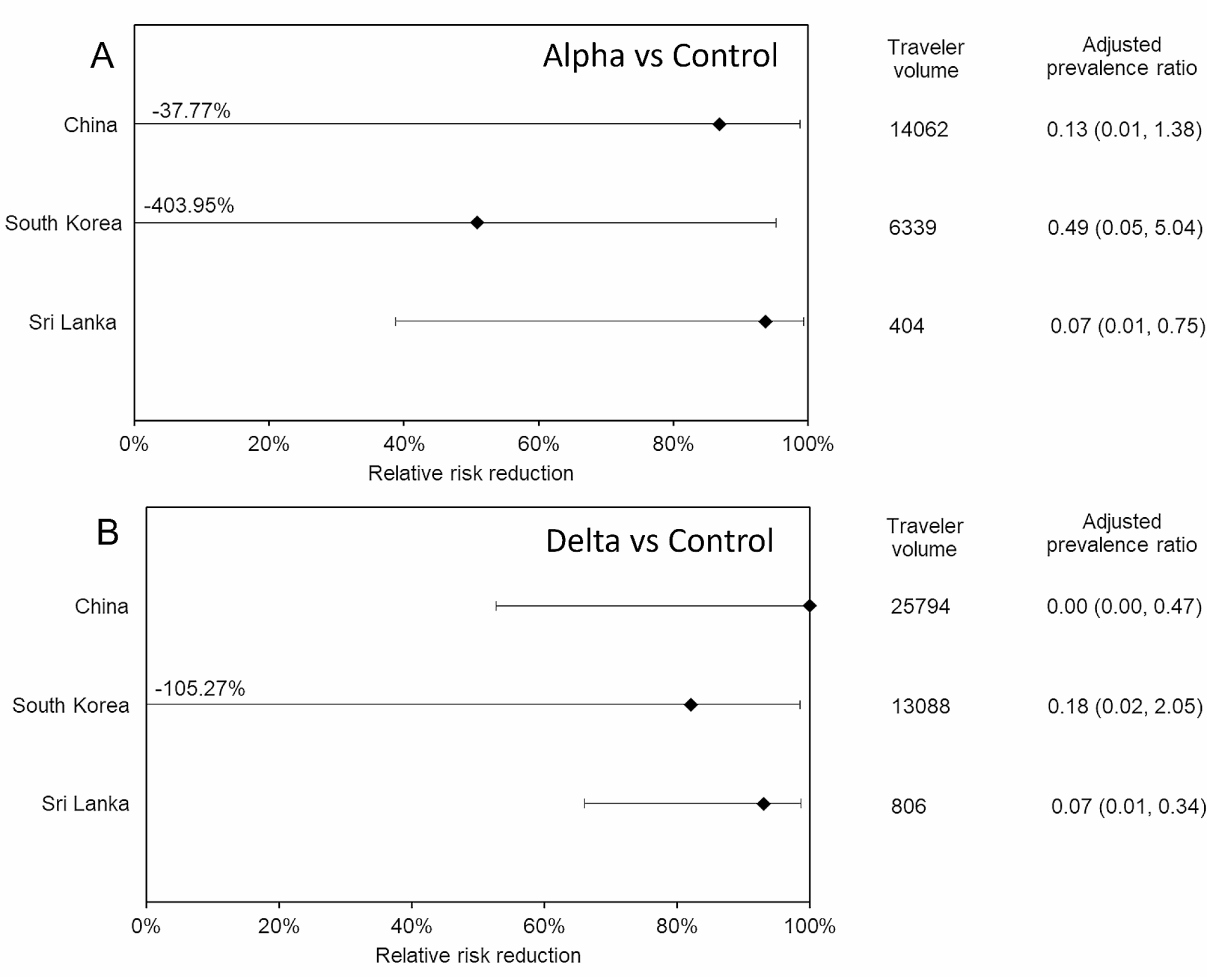
Relative risk reductions of COVID-19 among non-Japanese travelers arriving from Asian countries, 2020–21. Relative risk reductions during (**A**) the Alpha variant period and (**B**) the Delta variant period among non-Japanese travelers arriving from China, South Korea and Sri Lanka are shown. Numeric lower bounds are provided for specific countries with extremely small values, and the adjusted prevalence ratio column includes values within parentheses denoting the corresponding 95% confidence intervals derived from binomial distribution

Figure 4 presents the relative risk reduction among foreign travelers arriving from China, South Korea, and Sri Lanka. Comparing the Alpha variant period against the control period (Fig. 4A), the relative risk reductions in China, South Korea and Sri Lanka were 86.9% (95% CI -37.8–98.8), 50.9% (95% CI -404.0–95.2), and 93.7% (95% CI 38.8–99.4), respectively. During the Delta variant period (Fig. 4B), the relative risk reductions in China, South Korea and Sri Lanka were 100% (95% CI 52.7–100), 82.0% (95% CI -105.3–98.4) and 93.1% (95% CI 66.0–98.6), respectively. During the Alpha variant period, the lower 95% CI for China crossed 0%, and for both periods, the lower 95% CI for South Korea also crossed 0%, and thus, the finding cannot be overexaggerated.

## Discussion

The present study evaluated the effectiveness of the exit screening policies for COVID-19 among Japanese and non-Japanese travelers arriving from Asian and Pacific countries using entry screening data in Japan. We defined three time periods, (i) a control period without exit screening, (ii) the Alpha variant dominated period in the presence of exit screening, and (iii) the Delta variant dominated period in the presence of exit screening, comparing origin-incidence adjusted relative risks of the Alpha and Delta variant periods against the control period. The adjusted relative risk decreased well below the value of one during the Alpha variant period, and that during Delta variant period was also generally below one, except for travelers from India and Myanmar. Via exit screening, the greatest relative risk reduction was seen in high-income countries during both the Alpha and Delta variant periods. Upper-middle-income countries also experienced substantial risk reduction, but lower-middle-income countries were not associated with a positive impact of exit screening during the Delta variant period.

To our knowledge, the present study is the first to evaluate the effectiveness of exit screening that is carried out in origin countries, using entry screening results in Japan as the outcome measure. The relative risk reduction of more than 50% for both high-income and upper-middle-income countries is consistent with our foregoing evaluation that focused on the United Kingdom (Liu et al., submitted); using not only entry screening data but also Office for National Statistics COVID-19 infection survey data, the exit screening in the UK was estimated to have reduced the risk of positivity in Japan by 59%. What is notable is that (i) risk reduction via exit screening became more challenging during the Delta variant period than the Alpha variant period, and (ii) depending on the origin country where exit screening was conducted, the risk reduction would be highly variable. As for (i), an elevated transmissibility (and perhaps also a shorter incubation period) of the Delta variant compared with the Alpha variant [21–24] may partly explain this difficulty. With respect to (ii), the South Asian countries experienced the largest-ever epidemic caused by the Delta variant [25–27], most notably in India [19, 28–30], and there were substantial number of passengers presenting pre-departure negative test certificates that did not meet testing quality standards; moreover, there were even reports of falsification of pre-departure certificates [31, 32]. Countries with high prevalence tended to yield increased relative risks (i.e. lower effectiveness of exit screening), while countries with low prevalence tended to yield lower relative risks.

How are our estimates of exit screening effectiveness compared against other published evidence? The present study used empirically observed entry screening data collected at airports in Japan, and from January 2021, the country required all travelers to undertake exit screening via RT-PCR testing conducted within 72 h prior to departure. Preceding simulation or epidemiological studies have investigated the optimal timing for exit screening using RT-PCR or antigen testing. Kiang et al. [9] suggested PCR testing within 3 days of departure, coupled with a post-quarantine test upon arrival, as the most effective strategy for reducing onward transmission. Via a modelling approach, Johansson et al. [10] found that testing a day before arrival resulted in a risk reduction of 17–35%, while testing 3 days before arrival yielded a 5–13% reduction in the risk of introduction. Chad et al. [11] showed that delays up to 72 h in obtaining RT-PCR test results led to a high probability (39.2%) of onward transmission, with a modest 4.5% reduction in expected post-arrival transmission. Bart et al. [12] demonstrated a 52% lower likelihood of positive post-arrival test results when pre-departure COVID-19 testing occurred within a day. Additionally, Clifford et al. [13] reported that testing a day before travel was more effective in reducing post-arrival transmission. These findings underscore the importance of tailored testing approaches based on real-world dynamics and transmission probabilities, offering insights for public health strategies. The present study, revealing decreased effectiveness in the Delta period, suggests that for the Japanese government and other public health agencies, the timing for travelers undergoing exit screening may need to be shortened when a more transmissible variant emerges, e.g., within 48 h. Moreover, the method of testing may also be flexible, allowing for the adoption of antigen testing.

The relative risk reduction that we estimated demonstrated the performance of exit screening, but that finding was positively associated with income levels. A critical shortcoming of this finding is that the quality assurance of exit screening must be made to ensure the effectiveness via a validated testing procedure; this key issue may be deemed problematic when the testing situation in the country of origin is complicated by local factors. For instance, one of the lower-middle-income countries, India, experienced an extremely large increase in the epidemic dominated by the Delta variant, and both the testing capacity and healthcare system itself were likely overwhelmed by massive number of severe cases [33, 34]. Of course, other explanations cannot be excluded. Although high-income countries yielded the greatest relative risk reduction, these countries included Australia, New Zealand and Singapore which had zero-COVID policies during the time period of our analysis, and the lowered risk during the Alpha and Delta variant periods could be explained by the heightened level of control in those countries [35–37]. The same applies to non-Japanese arriving from China and South Korea with substantial travel volume. They showed a decreased risk of infection, but the control program was strengthened as the variant of concern began to be widespread, and their success in controlling the outbreak with substantial surveillance capabilities at the origin could explain the observed risk reduction [38–41]. Of course, low effectiveness of exit screening among low income countries could also be explained by other factors, e.g. limited capacity of surveillance and low ascertainment rate.

Three technical limitations of the present study should be discussed. First, our primary results were derived among Japanese travelers returning to Japan. This group was selected because of data availability and also the absence of exit screening policy during the control period. If Japanese people tended to strictly undertake exit screening compared with other nations, we may have overestimated the effectiveness of exit screening. Second, individuals who are likely to travel are predominantly healthy, suggesting the lower risk propensity of severity among travelers compared to the general population. This may elevate the risk of infection when free travel is allowed, but under the suppression policies that greatly reduced incidence, healthy travelers may have been less likely to be infected compared with others. In fact, Kucharski et al. [42] demonstrated that positivity at entry screening is likely to be lower than the prevalence at the origin. Thus, using entry screening data as the outcome may have already been relying on a substantially reduced risk of infection, and moreover, our estimate may be quite biased (perhaps overestimated) compared with the policy applied to the entire population. Third, the study did not handle the time period in which the Omicron variant was predominant. Changing characteristics of the epidemiology due to variant replacement and overwhelming pressure of case load demand can alter the ascertainment and also the effectiveness of exit screening. Our results were restricted to 2020–21, and additional insights must be gained by exploring datasets that cover the period after the emergence of Omicron variant.

## Conclusions

Despite these limitations, we believe that the present study was successful in evaluating the effectiveness of exit screening policy among travelers arriving from Asian and Pacific countries using entry screening data in Japan. The adjusted relative risk decreased well below the value of one during the Alpha variant period, and that during the Delta variant period was also mostly below one except for India and Myanmar. Via exit screening, the greatest relative risk reduction was seen in high-income countries during both the Alpha and Delta variant periods. Upper-middle-income countries also experienced substantial reductions, but lower-middle-income countries did not reveal a positive impact of exit screening during Delta variant period.

### Electronic supplementary material

Below is the link to the electronic supplementary material.

Supplementary Material 1

Supplementary Material 2

## Data Availability

Airport entry screening data for Japanese and non-Japanese passengers are publicly available on the website of the Ministry of Health, Labour and Welfare [16] and are presented in the Additional Data file. The data sources are acknowledged for their contributions, and we adhered to their respective data-sharing policies and terms of use.

## Abbreviations

CI: Confidence interval
COVID-19: coronavirus disease 2019
RT-PCR: real-time reverse transcription-polymerase chain reaction
