# Supplementary material for: Evaluation of the exit screening policy among travelers arriving from Asian and pacific nations
Source: BMC Infect Dis. 2024 May 2;24:464. doi: 10.1186/s12879-024-09327-8 (PMC11067274; doi:10.1186/s12879-024-09327-8)

**Additional File 1:** Additional results

**Additional data.** Data on airport entry screening positivity by country of origin.

**Figure S1. Exit screening policy timeline among foreign travelers from Asian and Pacific countries.**

The blue bar indicates the period during which exit screening was required. Because exit screening was imposed from 1 September 2020, we were not able to define a control period for the majority of foreign travelers, and thus we focused on Japanese travelers arriving from these countries as they were not mandated to submit the certificate of negative testing by January 2021.

**
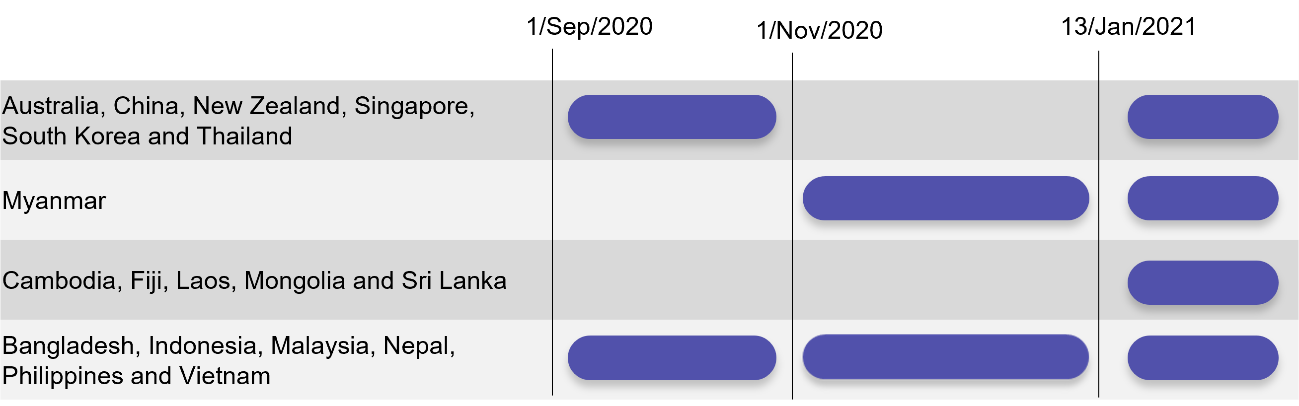
**

**Figure S2. Sensitivity analysis of the relative risk reduction among Japanese travelers returning from nine countries during the Alpha period versus control periods from 9 weeks to 15 weeks.**

The yellow square denotes a 9-week control period, the blue diamond represents the baseline 12-week control period, and the red circle signifies a 15-week control period. Within the adjusted prevalence ratio column, values in parentheses denote the respective 95% confidence intervals derived from a binomial distribution.


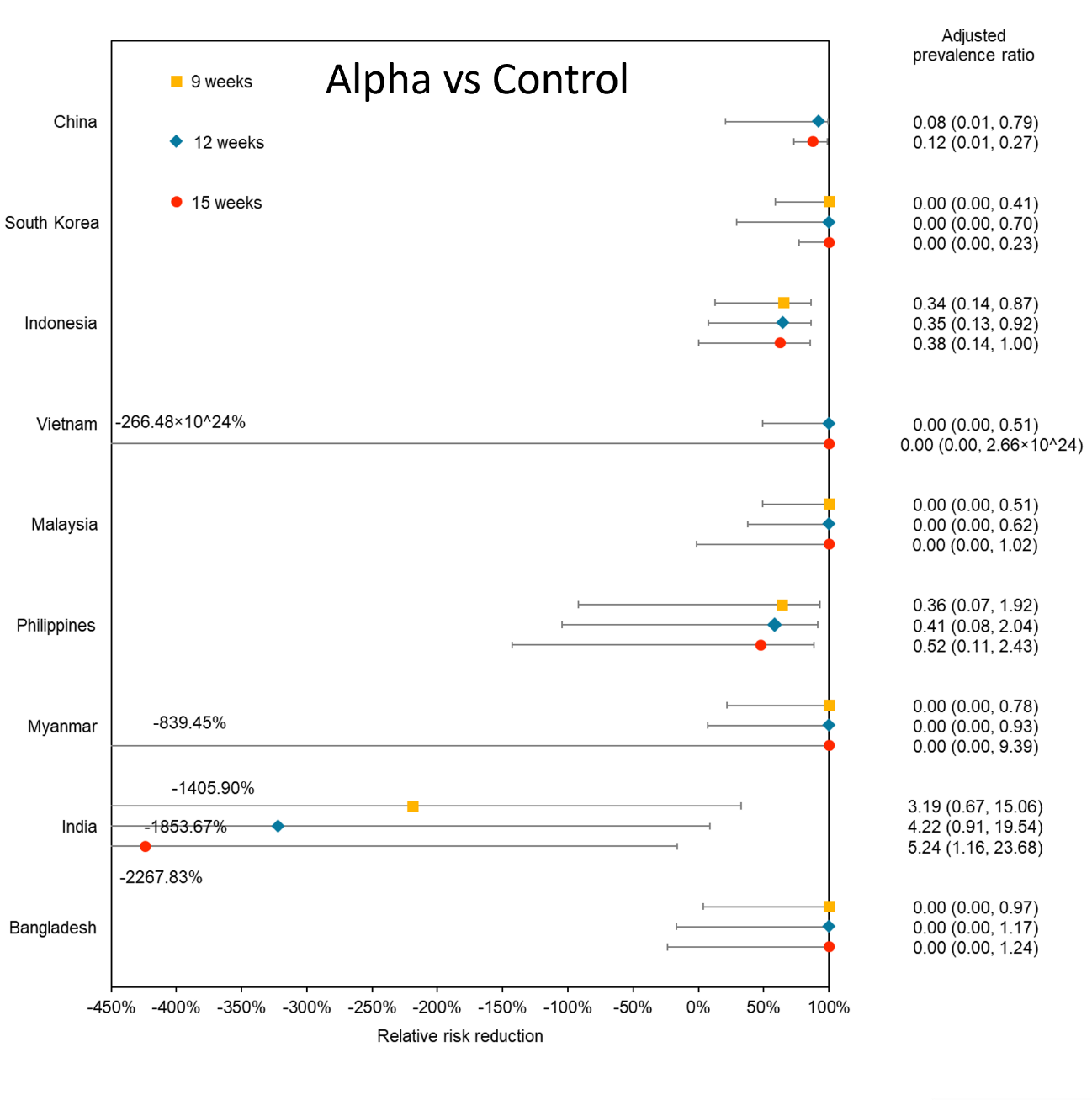


**Figure S3. Sensitivity analysis of the relative risk reduction among Japanese travelers returning from nine countries during the Delta period versus control periods from 9 weeks to 15 weeks.**

The yellow square denotes a 9-week control period, the blue diamond represents the baseline 12-week control period, and the red circle signifies a 15-week control period. Within the adjusted prevalence ratio column, values in parentheses denote the respective 95% confidence intervals derived from a binomial distribution.


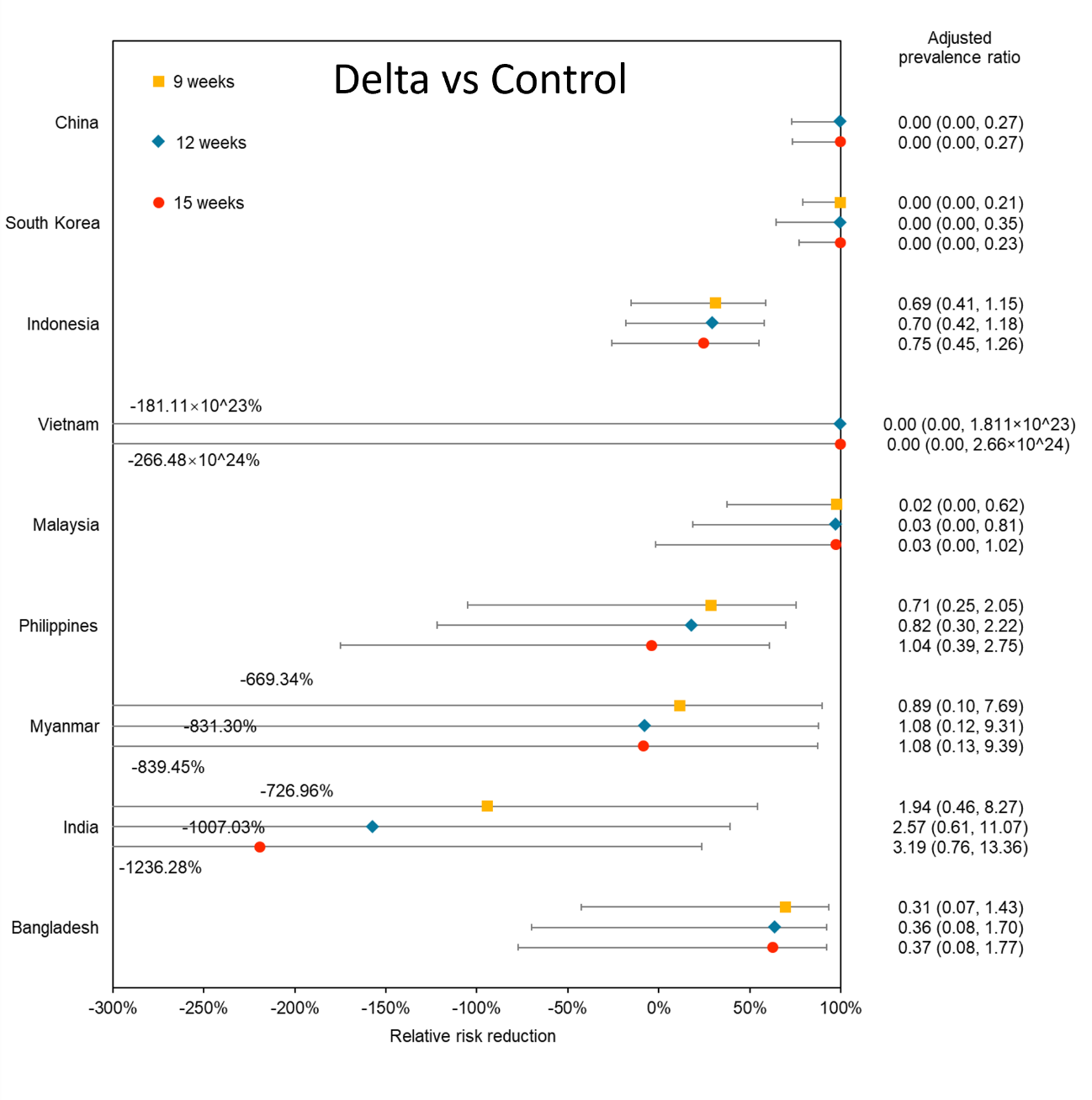


**Figure S4. Relative risks of COVID-19 among Japanese travelers returning from nine countries during the Alpha and Delta periods compared with the 11-week control period, 2020–21.**

For both periods, the dashed line represents a relative risk value of 1, implying no indication of the effectiveness of exit screening. Numerical values on the whisker denote upper bound values among indivdual countries. In the unadjusted prevalence ratio columns, values inside parentheses denote the corresponding 95% confidence intervals derived from the binomial distribution.


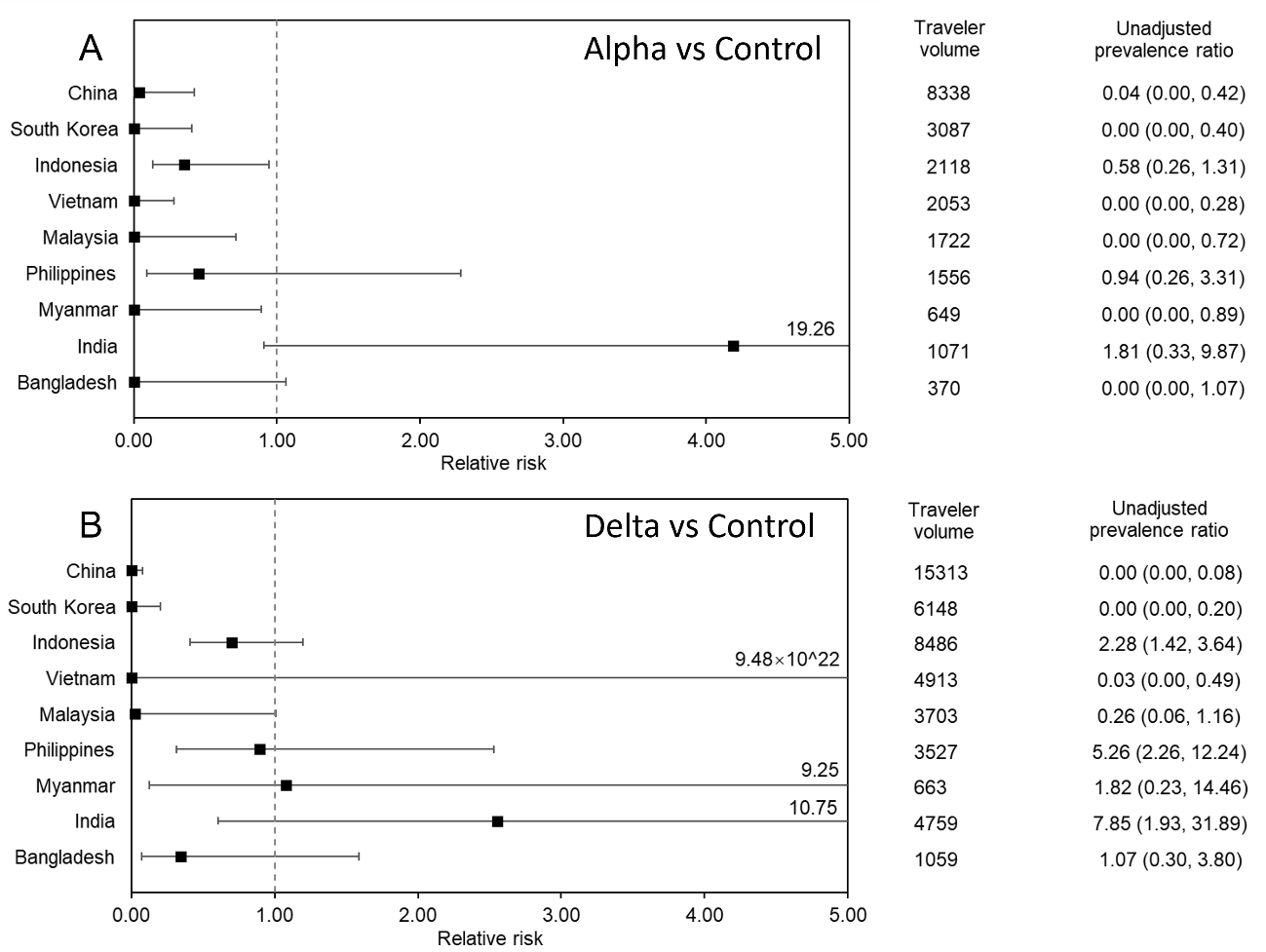

Supplement: Supplementary file 2 — Supplementary Material 2 [file 12879_2024_9327_MOESM2_ESM.docx]
